# The Treponema Pallidum

**Published:** 1913-03

**Authors:** R. H. L. Bibb


### EDITORIAL DEPARTMENT.

**DR. F. E. DANIEL, Editor** **DR. R. H. L. BIBB, Associate Editor**

DEPARTMENTS { EUGENICS: Drs. M. Duggan and T. Y. Hull.

WOMAN'S DEPARTMENT: Mrs. F. E. Daniel.

### The Treponema Pallidum.

Working in the John Howard McFadden Researches Department of the Lister Institute, E. H. Ross publishes, in the *British Medical Journal* for December 14, 1912, some highly interesting and important discoveries, which he illustrates with a polychrome plate, relating to the *treponema pallidum,* which go far to prove, if, indeed, they do not prove, that this somewhat ubiquitous little spiral is a *microgamete* in the life history of a *protozoan* which he calls *lymphocytozoon,* a parasite to be found in the lesions and embedded within the mononuclear cells of all animals infected with syphilis, just as is the flagellum of the malarial parasite the *microgamete* of the malarial organism—the *microgametes* being the male, and the *macrogametes* the female elements on which the parasites depend for their perpetuation.

According to Ross, a homologous protozoan is to be found in the blood of the guinea pig, the *lymphocytozoon cabayae,* in the vesiculae seminalis of the common earthworm, the *lymphocytozoon lumbrici,* in the rabbit, the *lymphocytozoon leporis,* and in rats, the *lymphocytozoon muris,* which resemble each other very closely and in all their phases, each species having *gametes* which are absolutely indistinguishable from the *spirochaeta pallida* in any way whatever; and he claims that the pear-shaped, sometimes round, inclusion within the cytoplasm of the mononuclear leucocytes, and also free in the blood plasma, of syphilitics only, and from which he has seen the *spirochaeta pallida* spring, is the homologue of the *lymphocytozoon* known as Kurloff's bodies, which Cropper has demonstrated to be parasites—the *lymphocytozoon,* the macrogametes or female bodies of the animals mentioned above.

Ross declares that animals infected with the *lymphocytozoon*—man, rats, guinea pigs and rabbits—show lesions identical in every particular, that are amenable to arsenical and mercurial compounds, and that he has never once failed, in two hundred consecutive cases of human syphilis, to demonstrate the *lymphocytozoon pallidum* in the blood and lesions of the syphilitics.

On account of the striking resemblance between the *lymphocytozoon pallidum* and the *lymphocytozoon cabayae* and the *lymphocytozoon leporis,* Ross suggests that they might produce a mild infection in man and thus modify human syphilis, as vaccination with the *cytoryctes vaccinae*—the supposed cause of vaccinia—modifies the effects of the *cytoryctes variolae*—the supposed germ of smallpox, discoveries of Guarnieri—and prevents the disease entirely or converts it into a harmless varioloid.

Jennings and Moolgavker—same journal—confirm Ross' claim as to the finding of the *lymphocytozoon* in cases of human syphilis as well as to its development into a spiral typical of the *treponema pallidum,* and illustrate their articles with wood engravings, which show the complete evolution of the parasite.

An admirable opportunity is offered to Noguchi and to Baeslack, who are engaged in experimental culture of the *treponema pallidum,* to refute or confirm the pretentions of Ross, as well as to throw much additional light on this very important subject, whereby the profession may be able hereafter to make a positive diagnosis of lues, in obscure cases, without resort to the cumbersome and sometimes misleading Wassermann reaction.

These discoveries—the importance and influence of which it would be exceedingly difficult to forecast at present—were made by vitro staining with Unna's polychrome methylene blue and a specially prepared jelly or culture medium, which permitted the investigators to directly witness every cycle of the parasite's evolution, from segmentation of the *lymphocytozoon* to the full-fledged spirillum. The process is simple and easy to apply, but is too lengthy for this writing. Directions for applying it will be sent, with much pleasure, by the writer, to anyone wishing to use it.

R. H. L. Bibb.

